# Study on the Carbonation Behavior of Steel Slag in the SiC-K_2_SiO_3_ System Assisted by Microwave Heating

**DOI:** 10.3390/ma19132701

**Published:** 2026-06-23

**Authors:** Wei Long, Wenxiao Fu, Wenming Jiang

**Affiliations:** 1 School of Materials and Chemical Engineering, Hubei University of Technology, Wuhan 430068, China; maillong1982@126.com (W.L.); fuwxxx@163.com (W.F.); 2 Key Laboratory of Green Materials for Light Industry of Hubei Provincial, Wuhan 430068, China; 3 Hubei Engineering Laboratory of Automotive Lightweight Materials and Processing, Wuhan 430068, China; 4 State Key Laboratory of Materials Processing and Die & Mould Technology, School of Materials Science and Engineering, Huazhong University of Science and Technology, Wuhan 430074, China

**Keywords:** steel slag, carbonation, microwave heating, alkaline activator

## Abstract

The steel industry is currently grappling with the dual environmental challenges of massive steel slag accumulation and carbon emissions. To address the limitations of traditional carbonation processes—namely slow reaction kinetics and insufficient mechanical properties—this study proposes a novel rapid carbonation enhancement method coupling microwave thermal field intensification, silicon carbide (SiC) physical absorption, and potassium silicate chemical activation. The effects of microwave heating parameters on the performance of carbonated steel slag blocks were systematically investigated. The results indicate a significant synergistic effect between the microwave thermal effect and the alkali-activated system. Under the conditions of a 0.14 liquid-to-solid ratio and microwave heating at 90 °C for 45 min, the compressive strength reached a peak of 48.82 MPa (a 44.7% increase over the conventional treatment group). Microstructural characterization revealed the reinforcement mechanism: the introduction of SiC and potassium silicate solution (K_2_SiO_3_) under microwave heating promotes a denser distribution of carbonation products. Synchronized with alkali activation, this effect promotes the in-situ growth of dense calcite crystals within a gel network, thereby significantly optimizing the pore structure (e.g., reducing the average pore size to 43 nm), and enhancing strength through synergistic effects. This research is subject to further energy and life-cycle assessments, and this approach holds potential for CO_2_ mineralization and the recycling of steel slag.

## 1. Introduction

Steel slag (SS), a byproduct originating from the pyrometallurgical processing of iron and steel, exceeds 100 million tons annually. Long-term stockpiling not only occupies land resources but also poses severe risks of air pollution and human health hazards due to heavy metal leaching and dust dispersion [1,2,3,4,5]. Currently, China produces approximately 100 million tons of steel slag annually, with stockpiles exceeding 1 billion tons [6], yet the comprehensive utilization rate remains only about 30% [7,8]. Therefore, developing an efficient and environmentally friendly method for treating and utilizing steel slag is imperative, particularly since its cement-like composition [9,10] makes it a viable raw material for building products. Nonetheless, the high concentrations of free lime (f-CaO) and periclase (f-MgO) often trigger volumetric instability and expansion-induced fracturing, which significantly limits the material’s large-scale application, resulting in extremely low utilization rates [4,11,12,13].

The steel industry is one of the main sources of global greenhouse gas emissions. In China, the steel industry alone accounts for approximately 15% of the nation’s total CO_2_ output [14]. CCUS (Carbon Capture, Utilization, and Storage) is considered the most effective strategy to significantly cut carbon emissions from steelmaking [15,16]. Through this approach, CO_2_ reacts with calcium-rich phases in steel slag to form stable calcium carbonate (CaCO_3_), achieving permanent carbon storage. This process not only reduces carbon dioxide emissions but also enables the recycling of solid waste [17,18,19,20,21].

In recent years, numerous researchers have made progress in the field of carbonation curing of steel slag, revealing the influence of various conditions such as slag particle size, temperature, CO_2_ pressure, and carbonation duration on the carbonation outcomes [22,23,24,25,26,27,28,29,30]. For instance, Huigen et al. [31] observed that when steel slag powder underwent suspension carbonation at 19 bar CO_2_ pressure and 100 °C for 30 min, 74 wt% of the CO_2_ in the slag was fixed within the carbonation products. This accelerated the carbonation process, enhancing its potential for application in construction materials. Bodor et al. [32] observed that carbonizing steel slag aggregates at 90 °C for 2 h achieved acceptable volume stability, enabling substitution of up to 50% of natural aggregates in concrete; Mahoutian et al. [33] indicated that carbonated steel slag binder achieved a compressive strength of 26.7 MPa after 24 h of carbonation. Tian et al. [34] concluded that temperature is the decisive factor governing direct gas–solid carbonation processes.

Conventional carbonation processes for steel slag often suffer from notoriously slow reaction kinetics and yield products with inadequate mechanical strength. Microwave heating, specifically, demonstrates several merits, such as bulk thermal distribution, targeted heating, and optimized power utilization, which may accelerate the kinetics of carbonation reactions. Currently, microwave heating is also applied in materials science [35,36,37,38,39], but the mechanism influencing the carbonation curing of steel slag under microwave heating remains unclear. Additionally, studies indicate that the hydration process of alkali-activated steel slag resembles that of cement. Peng et al. [40] utilized water glass to activate the cementitious properties of steel slag and slag, and found that the strength of the resulting cementitious materials increased after alkali activation. Zhou et al. [41] employed steel slag as the primary raw material, with blast furnace slag as a modifier, and added water glass with a modulus of 1.5 and 4% Na_2_O for alkali activation. The results indicated that water glass refined the pore structure, significantly enhancing the material’s mechanical properties.

Although existing studies have explored the effects of temperature, pressure, and activators on carbonation performance, traditional processes generally suffer from long production cycles, low early-stage strength, and incomplete reactions caused by carbonation barriers. Due to its volumetric and selective heating characteristics, microwave heating has demonstrated exceptional energy efficiency in material synthesis and holds great potential to overcome the gradient limitations of traditional thermal conduction from a kinetic perspective. However, the response mechanism of steel slag carbonation under a microwave thermal field remains unclear. In particular, the dynamic coupling mechanism between the physical thermal field (microwaves) and chemical activation (alkali activators) lacks further investigation.

In this study, a potassium silicate solution (K_2_SiO_3_) with a modulus of 1.04 was employed as the alkali activator, integrated with microwave heating and silicon carbide (SiC) as a microwave absorption enhancer. The evolution of steel slag compacts within the microwave-assisted carbonation system was systematically investigated. In combination with various microscopic characterization techniques, the reinforcement mechanism was elucidated. Specifically, the microwave-induced crystal growth patterns and pore structure reconfiguration mechanisms were highlighted. The findings of this research provide theoretical support and data references for industrial-scale microwave-assisted carbonation and carbon sequestration technologies.

## 2. Experimental

### 2.1. Materials

The raw materials used in this experiment were BOF steel slag powder and silicon carbide powder. BOF steel slag powder (SS) and silicon carbide powder (SiC) were sourced from China Baowu Steel Group (Shanghai, China) and Jiangsu Yuante New Material Technology Co., Ltd. (Wuxi, Jiangsu, China), respectively. Prior to testing, the steel slag powder and silicon carbide powder were dried in a vacuum oven at 60 °C for 24 h to remove excess moisture.

The solution used was a potassium silicate solution (K_2_SiO_3_) prepared in-house with a modulus of 1.04 (SiO_2_/K_2_O). The density of steel slag powder was measured at 3.541 g/cm^3^ using the pycnometer method, while that of silicon carbide powder was 3.215 g/cm^3^. The specific surface area of steel slag powder was determined to be 362 m^2^/kg using a Quanta Chrome fully automatic analyzer (Anton Paar QuantaTec Inc., Boynton Beach, FL, USA). The chemical composition of steel slag powder and silicon carbide powder was analyzed by X-ray fluorescence (XRF), with the results shown in Table 1.

**Figure 1:**
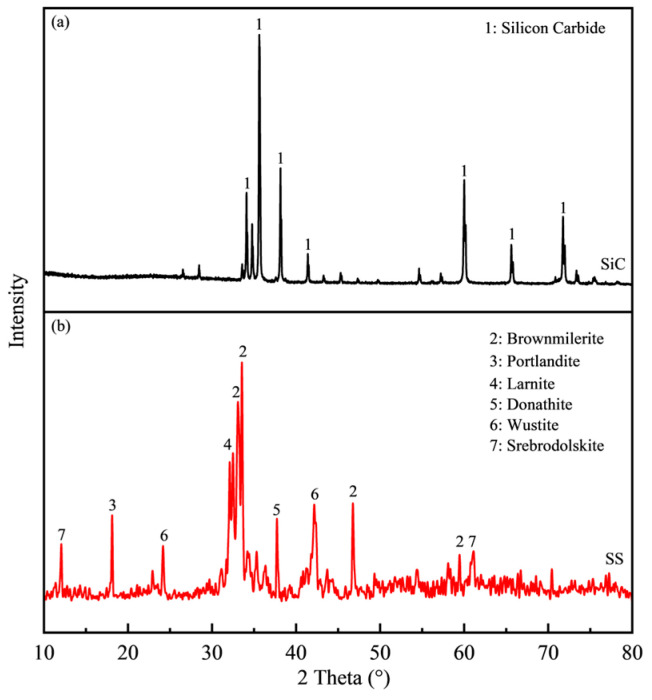
Mineral composition of silicon carbide (**a**) powder and steel slag (**b**).

**Figure 2:**
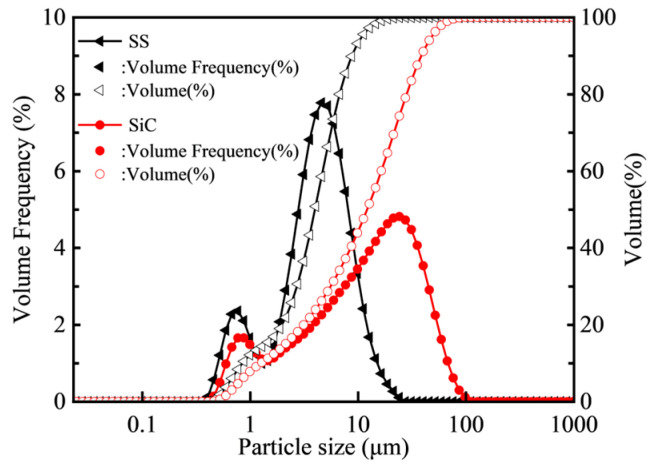
Particle size distribution of steel slag (SS) powder and silicon carbide (SiC).

The mineral phases of steel slag and silicon carbide were determined by X-ray diffraction analysis (Bruker AXS, Karlsruhe, Germany), with the results shown in Figure 1a,b. The primary minerals in steel slag include brownmillerite (Ca_4_F), portlandite (Ca(OH)_2_), larnite (Ca_2_SiO_4_), Donathite (Ca_2_MgSi_2_O_7_), and Srebrodolskite (Ca_2_Fe_2_O). The particle size distributions of silicon carbide and steel slag were determined using a Mastersizer 2000 laser particle size analyzer (Malvern Panalytical, Malvern, UK), as shown in Figure 2. Silicon carbide and steel slag exhibited particle size ranges of 0.405 µm to 24.1 µm and 0.46 µm to 98.1 µm, respectively.

### 2.2. Experimental Processes

Compaction degree is defined as the volume ratio of the steel slag sample to the whole specimen. To keep the total volume constant, the specimen height was fixed, and the compaction degree was computed using Equation (1) [42], following the method in [6]. This study uniformly mixed 98 wt% steel slag with 2 wt% silicon carbide, then the mixture was blended with potassium silicate (43.6°Bé) with a modulus of 1.04 (K_2_O/SiO_2_) or deionized water according to the required liquid-to-solid ratio (The ratio of the total mass of the solution to the mass of the solid, it consists mainly of K_2_O and SiO_2_). After thorough mixing, the resulting mixture was compacted using a pressing method. The mixture was loaded into a custom mold at the weight required 13.94 g for 55% compaction. After maintaining pressure for 120 s, the mold was removed to form cylindrical specimens with a diameter of 20 mm and a height of 20 mm. Five specimens were prepared for each mixture ratio in this study.

*Degree of compaction* = (*m*/*ρ*)/(*πD*^2^/4 × *h*) × 100% (1)

In the formula, *m* refers to the mass of the steel slag compact prior to carbonation, g; *ρ* indicates the density of the steel slag, g/cm^3^; *D* is the diameter of the compact, cm; *h* stands for the specimen’s height, measured in cm.

**Figure 3:**
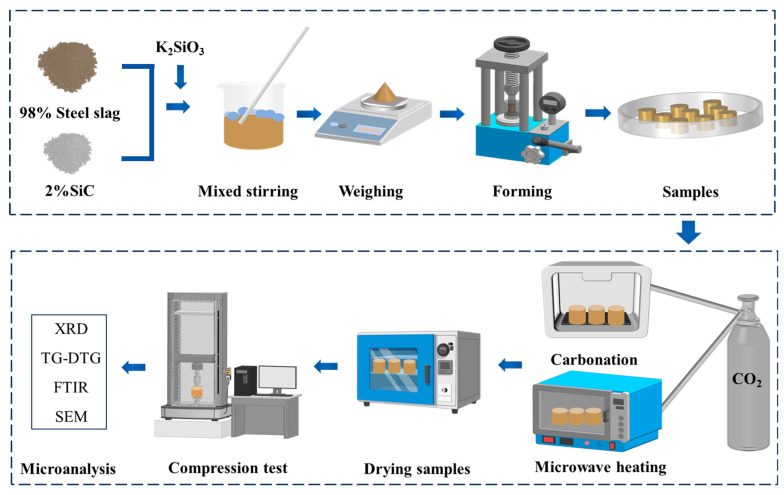
Sample preparation and processing flowchart.

Following the successful preparation of the specimens, carbonation was conducted at ambient temperature for 3 h under a CO_2_ purity of 99.9%, with a pressure of 0.1 MPa. Subsequently, the samples were subjected to microwave treatment. In this study, the microwave output power was fixed at 2.45 GHz with a heating power of 500 W. A segmented heating method was adopted: maintaining a constant temperature, reaching the target temperature through heating for 5–7 min, followed with a 2-min heating interval (Intermittent heating prevents heat damage). This cycle was repeated at fixed heating temperatures of 70 °C, 80 °C, and 90 °C. A temperature sensor was installed inside the microwave reactor (The measured temperature is the air temperature inside the microwave reactor). Heating durations were set at 15 min, 30 min, 45 min, and 60 min, as detailed in Table 2. During microwave treatment, CO_2_ was continuously fed into the microwave reactor (FCMCR-3SX) (Gongyi Kerui Instrument Co., Ltd., Gongyi, China). After microwave processing, carbonation continued at ambient temperature until the reaction concluded at 9 h. After reaction completion, samples were dried in a vacuum drying oven at a precisely controlled temperature of 60 ± 1 °C for 12 h. The sample preparation and processing workflow is illustrated in Figure 3 (The K_2_SiO_3_ shown in the figure refers to a potassium silicate solution), clearly depicting the complete experimental procedure. 

### 2.3. Test Methods

#### 2.3.1. Compressive Strength and CO_2_ Uptake

Compressive strength was measured using an HBGYDX-100M microcomputer-controlled electronic universal testing machine (manufactured by Jinan Zhongluchang Testing Instrument (Jinan, China)). The instrument applied a load at a rate of 2 mm/min, and the final result for each group was the average of three specimens.

The CO_2_ absorption capacity of the samples was calculated using Formula (2) [43] to measure the degree of carbon dioxide absorption by the samples. The water collected from the carbonation vessel represents moisture lost from the compacts due to the exothermic carbonation reaction; this water is incorporated into the final mass calculation of the carbonated specimens. In practice, it condenses on the vessel walls and is gathered using absorbent paper. Mass_post-carbonated_ refers to the mass after vacuum drying at 60 ± 1 °C.

(2) $$CO_{2}uptake=\frac{{mass}_{post-carbonated}+{mass}_{collectedwater}-{mass}_{pre-carbonated}}{{mass}_{solid}}\times 100\%$$

#### 2.3.2. Composition and Microstructural Analysis

X-ray diffraction patterns were analyzed using X-ray diffraction technology (XRD, Empyrean, Malvern Panalytical, Almelo, The Netherlands) equipped with a Cu Kα radiation source to obtain the phase analysis of the samples. Experimental conditions were set at an operating voltage of 45 kV and a current of 40 mA, with a diffraction angle range of 10–70° scanned at a rate of 5° per minute.

A Czech TESCAN MIRA LMS scanning electron microscope (SEM) (TESCAN a.s., Brno, Czech Republic) was employed to conduct detailed observation and analysis of the surface and internal microstructure of steel slag samples before and after carbonation treatment, focusing on an internal region approximately 8 mm from the surface. Prior to SEM testing, samples underwent 180 s of gold sputtering using a Quorum SC7620 sputter coater (Quorum Technologies Ltd., Laughton, UK).

Fourier Transform Infrared (FTIR) analysis (Thermo Fisher Scientific, Waltham, MA, USA) was performed using a Nicolet 170SX instrument (Nicolet Instrument Corporation, Madison, WI, USA) to obtain information on molecular groups present in the samples. Using potassium bromide (KBr) for sample preparation, FTIR spectra were recorded across a wavenumber span of 4000 to 400 cm^−1^.

Pore volume analysis was meticulously performed using an automatic mercury porosimeter (Micromeritics AutoPore V9600) (Micromeritics Instrument Corporation, Norcross, GA, USA) to assess pore volume before and after microwave heating treatment. Pore structures (5 nm–350 µm) were analyzed following a 24 h vacuum-drying treatment at 60 °C to eliminate residual water.

Thermogravimetric-differential scanning calorimetry (TG-DSC) analysis was performed on selected samples using a NETZSCH TG-DSC instrument (model TG209F3) (NETZSCH-Gerätebau GmbH, Selb, Germany). During the experiment, the temperature range was set from 30 to 800 °C with a heating rate of 10 °C /min. To ensure experimental accuracy and reproducibility, nitrogen was used as the protective gas.

## 3. Results and Discussion

### 3.1. Analysis of the Synergistic Effect Between the Silicon Carbide Potassium Silicate System and Microwave Heating

**Figure 4:**
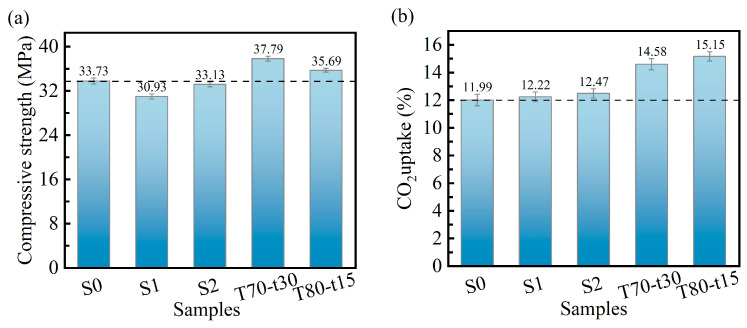
Comparison chart of compressive strength (**a**) and CO_2_ uptake (**b**) under different treatment conditions.

To investigate the synergistic effect between the introduction of the silicon carbide-potassium silicate system and microwave irradiation, this study designed and prepared control and experimental samples. Their compressive strength results are shown in Figure 4. The specific sample designs are as follows: S0 (water-only carbonation at room temperature for 9 h); S1 and S2 (based on S0, subjected to microwave heating at 70 °C for 30 min and 80 °C for 15 min during the third hour of carbonation, respectively). T70-t30 and T80-t15 represent the microwave treatment groups identical to S1 and S2 after introducing the silicon carbide-potassium silicate system.

As shown in Figure 4a, regarding compressive strength, in a pure water system, the contribution of microwave heating to strength does exhibit differences: compared to S0, the strength of S1 (70 °C) decreases by 8.3%, which may be attributed to rapid microwave-induced water evaporation leading to early-stage loosening of the structure, where the filling effect from carbonation reaction is insufficient to compensate for this physical damage. In contrast, the strength of S2 (80 °C) remains almost equivalent to that of S0, indicating that the elevated temperature accelerates the carbonation rate, thereby offsetting the negative impact of thermal stress.

The data indicate that although T80-t15 achieves the highest CO_2_ uptake, its compressive strength is lower than that of T70-t30. This suggests that the total amount of carbonation products is not the sole determinant of strength; rather, their distribution morphology and the synergistic network formed with alkali-activated products are more critical. Under the T70-t30 condition, the mild reaction rate facilitates the interwoven growth of gel phases and carbonate crystals, resulting in a denser skeleton. In contrast, the rapid carbonation under T80-t15 may lead to excessive product accumulation on the surface, forming an uneven ‘shell layer’ that restricts further internal structural optimization.

The reinforcement mechanism can be summarized as follows: first, as an efficient microwave-absorbing agent, SiC converts microwave energy into in situ thermal energy via the dielectric loss mechanism, achieving rapid and uniform temperature rise throughout the matrix. Second, the potassium silicate solution (K_2_SiO_3_), serving as the reaction medium, provides abundant silicate ions that promote the formation of hydration products. It indicates that there may be a synergistic effect between “SiC thermal induction + potassium silicate chemical activation”. The varying impacts of different microwave heating conditions—as evidenced by the comparison between T70-t30 and T80-t15—demonstrate that microwave heating parameters influence the carbonation performance of steel slag to different degrees.

### 3.2. Effect of Liquid-Solid Ratio on the Performance of Carbon Steel Slag Compacts

**Figure 5:**
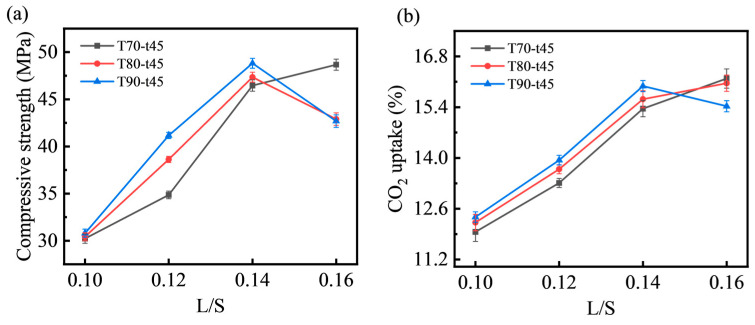
Comparison chart of compressive strength (**a**) and CO_2_ uptake (**b**) under different liquid-solid ratios.

To investigate the influence of the liquid-to-solid (L/S) ratio on the performance of carbonated steel slag compacts, compressive strength and CO_2_ uptake tests were conducted on specimens with L/S ratios of 0.1, 0.12, 0.14, and 0.16. The results are presented in Figure 5. The experiments were performed at heating temperatures of 70 °C, 80 °C, and 90 °C, with a constant heating duration of 45 min.

As shown in Figure 5, the compressive strength exhibits a significant upward trend as the L/S ratio increases from 0.10 to 0.14, reaching its peak at L/S = 0.14. It is speculated that an appropriate amount of water, serving as a medium for ion migration, may facilitate the dissolution of precursor materials in the alkaline solution to produce [SO_4_]^4−^ and metal cations, and promote the polycondensation of the generated [SO_4_]^4−^ with cations such as Ca^2+^ and K^+^ in the solution, potentially forming reaction products like C-S-H/K-S-H. These reaction products may have a synergistic strengthening effect with the calcite crystals formed by carbonation, which is considered a crucial reason for the significant improvement in early strength. When the L/S ratio reaches 0.16, the compressive strength decreases, and the CO_2_ uptake also shows a slight declining trend. This performance degradation may be attributed to a “water-locking effect”: excess water fills the capillary pores between steel slag particles. Since the diffusion coefficient of gas in liquid is much lower than that in the gas phase, an overly thick water film may hinder the diffusion of CO_2_ into the deeper layers, resulting in a slight decrease in performance.

Considering both mechanical properties and carbon sequestration efficiency, this study identified 0.14 as the optimal L/S ratio. At this ratio, water is considered to adequately meet the requirements for ion exchange and migration without excessively blocking gas channels, thereby achieving a reasonable balance between carbonation reaction kinetics and structural densification to some extent.

### 3.3. Effect of Microwave Heating Parameters on the Properties of Carbonated Steel Slag Pressed Blocks

**Figure 6:**
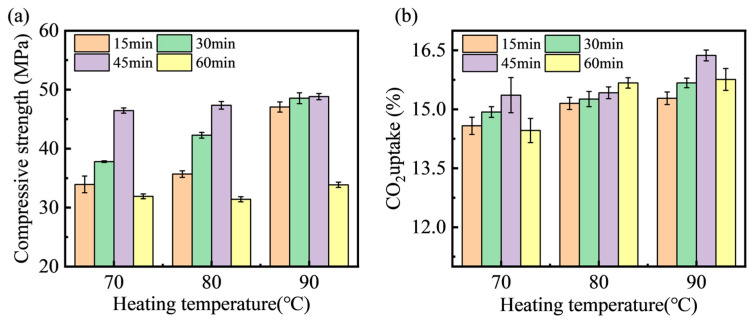
Comparison chart of compressive strength (**a**) and CO_2_ uptake (**b**) under different microwave treatment conditions.

To further systematically investigate the influence of microwave heating parameters on the performance of carbonated steel slag compacts, the specimens were subjected to different microwave temperatures and durations after 3 h of carbonation. Upon the completion of 9 h of carbonation, compressive strength and CO_2_ uptake tests were conducted. Figure 6 presents a comparison of the compressive strength and CO_2_ uptake for steel slag samples subjected to various microwave parameters following the initial 3 h of carbonation. The optimal liquid-to-solid (L/S) ratio of 0.14 was selected, with heating temperatures set at 70 °C, 80 °C, and 90 °C, and heating durations of 15 min, 30 min, 45 min, and 60 min.

As shown in Figure 6, microwave heating significantly enhances both the mechanical properties and carbon sequestration capacity of carbonated steel slag specimens; however, the enhancement effect depends on the synergistic regulation of temperature and duration. At a constant heating duration, the compressive strength increases monotonically with rising microwave temperatures. For instance, at 30 min, the compressive strength grew from 35.96 MPa at 70 °C to 47.06 MPa at 90 °C, representing a 30.9% increase. According to the Arrhenius law, an increase in temperature raises the proportion of molecules with sufficient energy to overcome the activation energy barrier, thereby significantly increasing the reaction rate constant. Under the microwave field, the added SiC acts as a high-loss medium that rapidly converts microwave energy into thermal energy, forming “hot spots” at the steel slag particle interfaces. This localized high temperature accelerates the dissolution of Ca^2+^ and the decomposition of phases within the slag, while simultaneously promoting the nucleation of CaCO_3_, thereby generating a large volume of calcite crystals within a short period.

At a constant heating temperature, the compressive strength exhibits a “threshold effect”, initially increasing and then decreasing with time. For example, at 80 °C, as the duration extended from 15 min to 45 min, the compressive strength grew continuously from 35.69 MPa to 47.35 MPa (a 32.7% increase); however, when the duration reached 60 min, the strength decreased to 31.41 MPa, indicating a “selective overheating” phenomenon where the promotional effect weakened. Despite the sharp decline in strength for the 60 min group, the CO_2_ uptake remained at a high level, suggesting that the loss of strength resulted from physical structural damage rather than the cessation of chemical reactions.

This degradation is attributed to the “inside-out” volumetric heating characteristic of microwaves. Under prolonged high-power microwave induction, the internal moisture of the specimen vaporizes rapidly. If the resulting vapor pressure exceeds the tensile strength of the early-stage gel structure, micro-cracks are induced within the material. Furthermore, the mismatched thermal expansion coefficients between the steel slag particles, SiC, and the generated CaCO_3_ crystals lead to significant thermal stress mismatch at the interfaces during excessive heat accumulation. This disrupts the gel network formed by potassium silicate, resulting in a loosened structure.

In summary, microwave heating exerts a significant catalytic effect on steel slag carbonation, yet there is a distinct “time window”. This study identifies 90 °C for 45 min as the optimal processing point to balance carbonation depth and structural integrity. Beyond this limit, microwave-induced thermo-physical damage offsets the reinforcement provided by chemical products.

### 3.4. Evolutionary Pattern of Strength Development with Carbonation Time of Steel Slag After Microwave Heating

**Figure 7:**
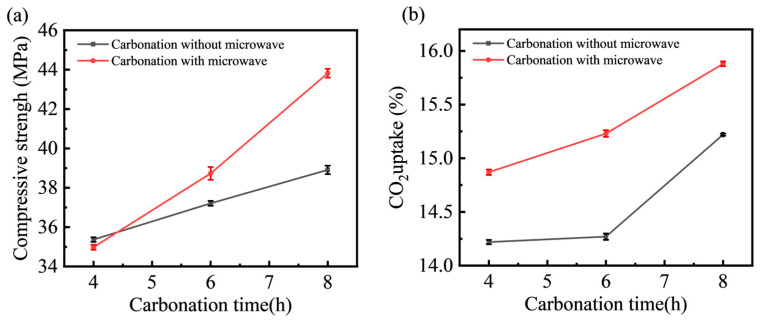
Compressive strength (**a**) and CO_2_ uptake (**b**) after microwave heating followed by carbonation at different durations.

To further investigate the effect of microwave heating on the carbonation process of steel slag, this study examined the properties of samples after carbonation for 4, 6, and 8 h following microwave heating intervention at the 3rd hour. A comparative experiment was designed to contrast samples heated under a non-microwave system with those in a SiC-K_2_SiO_3_ system, both heated at 90 °C for 45 min. The results are shown in Figure 7.

As shown in Figure 7a, during the continued carbonation process from 4 h to 8 h, the increase in intensity in the microwave-treated group was significantly higher than that in the room-temperature control group. At 8 h, the compressive strength of the microwave group reached 43.8 MPa, approximately 13% higher than the ambient group. As a high-loss auxiliary absorber, SiC generates a strong interfacial thermal effect within the microwave field. This localized high temperature maybe breaks through the dense carbonation barriers formed early on the steel slag surface, enhancing the ion diffusion rate. The environment, enriched with silicate ions and metal cations provided by the potassium silicate accelerates the dissolution of mineral phases under SiC-induced high temperatures. The dissolved Ca^2+^ and K^+^ ions combine with silicate ions to form high-density gel phases, which fill the spaces between the calcite crystals. These gel phases act as a “binder” consolidating the loose carbonation products into a dense, monolithic structure.

### 3.5. Microstructural Characterization and Mechanism Analysis

#### 3.5.1. Impact of Microwave Synergy on Pore Structure Reconfiguration

To elucidate the underlying mechanism by which the synergistic system enhances the carbonation performance of steel slag at the microscopic scale, various microstructural characterizations were performed on representative specimens.

**Figure 8:**
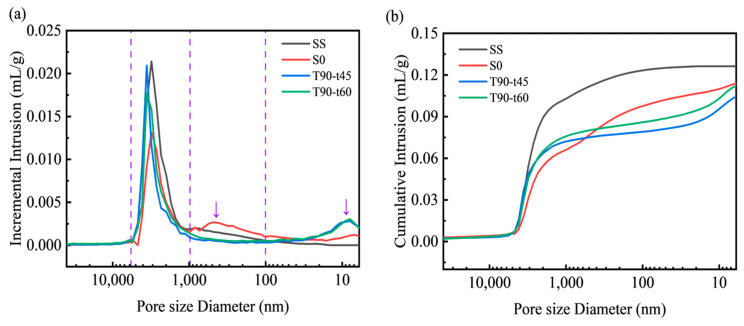
Pore size distribution (**a**) and cumulative intrusion (**b**) of samples under different treatment conditions.

Mercury Intrusion Porosimetry (MIP) was employed to determine the pore structure of the non-carbonated ambient-cured sample (SS), the 9-h ambient water-carbonation sample (S0), the optimal group (T90-t45), and the group exhibiting a decline in compressive strength (T90-t60). The evolution of incremental intrusion volume and cumulative intrusion volume with respect to pore diameter is illustrated in Figure 8.

As shown in Figure 8a, the untreated SS sample exhibits a broad and high primary intrusion peak in the 1000–10,000 nm range, reflecting the initial coarse capillary pores formed by the loose packing of steel slag particles. For the S0 group after 9 h of carbonation, the peak intensity in this range is significantly reduced, and a weak response appears within the 100–1000 nm range. This indicates that products such as CaCO_3_ generated by conventional carbonation fill some macropores to a certain extent, leading to a preliminary refinement of the pore size distribution.

In contrast, the T90-t45 group, incorporating the microwave synergistic system, demonstrates unique pore evolution characteristics. The sharp and regular intrusion peaks in the 1000–10,000 nm range for the T90-t45 group do not imply an increase in macropores; rather, they indicate that under microwave induction, the original disordered coarse pores are uniformly partitioned by a large volume of generated cementitious products and converted into closely packed, concentrated characteristic pores. Within the small pore size range of 10–100 nm, the response of the T90-t45 group is significantly enhanced. Conventional carbonation is often limited by the “carbonation barrier” formed by surface product layers, making it difficult for CO_2_ to diffuse into deeper layers. Lu et al. [44] demonstrated that for concrete, CO_2_ diffusion is difficult through pores smaller than approximately 20 nm.

However, the interfacial thermal effect generated by the synergy of microwave and SiC effectively alleviates the ‘diffusion barrier’ limitation in conventional carbonation processes, driving the in-situ precipitation of carbonation products within fine pores. This high-density and well-distributed pore network ensures continuous CO_2_ permeation paths in the later stages of the reaction and significantly strengthens the pore walls through the synergistic deposition of alkali-activated products and carbonation products.

However, considering the inherent limitations of MIP in characterizing complex matrices—such as the bias in pore size distribution caused by the ink-bottle effect, the influence of pore connectivity on mercury intrusion, and the potential interference from microcracks generated during sample preparation—this paper adopts a cautious approach when interpreting the evolution of pore structure. In addition to referencing changes in the average pore diameter, greater emphasis is placed on combining the decreasing trend in total porosity with the quantitative increase in macroscopic mechanical strength to comprehensively evaluate the degree of matrix densification.

The cumulative intrusion curves in Figure 8b confirm the densification trend of the system. The total cumulative intrusion volumes of the T90-t45 and T90-t60 groups are significantly lower than those of the SS and S0 groups, indicating that the synergistic system promotes a more thorough chemical reaction. The generated products achieve efficient filling of internal spaces, particularly for medium and small pores. Notably, the cumulative intrusion volume of T90-t60 rises slightly compared to T90-t45; this strongly corroborates the analysis of the “strength turning point” in Section 3.3: excessive microwave heating duration induces micro-cracks or thermal stress voids within the material, compromising structural density and leading to decreased mechanical performance.

Table 3 summarizes the porosity, bulk density, and apparent density of each sample group. The T90-t45 group exhibits the lowest average pore diameter and total porosity, elucidating the fundamental physical reason for its optimal compressive strength.

#### 3.5.2. Thermal Decomposition Behavior and Carbonation Degree Assessment

**Figure 9:**
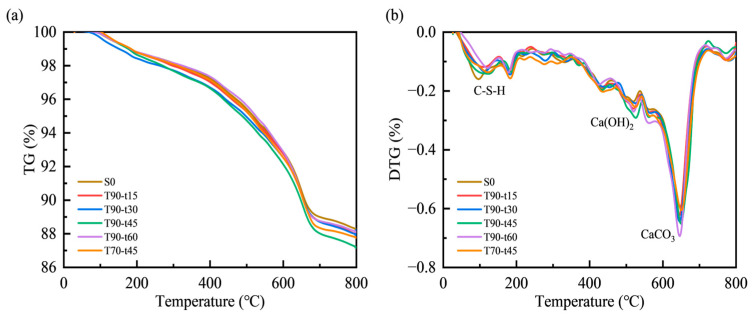
TG (**a**) and DTG (**b**) patterns of carbonated steel slag under different treatment conditions.

The thermal decomposition behavior of carbonated steel slag specimens under different treatment conditions was measured using a thermogravimetric analyzer (TGA) in an N_2_ atmosphere. The TG and DTG results are shown in Figure 9.

As shown in Figure 9, the TG and DTG curves of all samples exhibit similar multi-stage thermal degradation characteristics within the range of 30 °C to 800 °C, primarily corresponding to the dehydration of different water forms and the decomposition of carbonates [45,46].

The mass loss occurring between 50 °C and 200 °C is mainly attributed to the removal of physically adsorbed water and interlayer water from the gel-like phase formed during hydration [47]. The weight loss rate of the microwave-treated group in this interval is slightly higher than that of the ambient temperature group, suggesting that potassium silicate accelerates the reorganization and gelation of the silicate skeleton under the microwave thermal field. The weight loss peaks in the ranges of 400–500 °C and 600–800 °C are primarily due to the thermal decomposition of Ca(OH)_2_ present in the raw steel slag and of the main product of the carbonation reaction, calcium carbonate [45].

The microwave-induced effect demonstrates clear temperature and time dependence: as the heating temperature rises, the cumulative weight loss in the 600–800 °C range increases significantly, indicating that high temperatures enhance reaction kinetics and promote CO_2_ uptake. Regarding heating duration, the mass loss increases initially and then decreases, reaching a peak in the T90-t45 group. This is consistent with the compressive strength results. The results indicate that there may be a positive correlation between the degree of carbonation and the mechanical properties.

As the microwave heating temperature and duration (up to 45 min) increase, the CaCO_3_ decomposition peak becomes increasingly sharp and shifts toward higher temperatures. This suggests that microwave assistance not only increases the quantity of reaction products but also optimizes the crystalline development of calcite, thereby enhancing its stability.

The T90-t45 group exhibits the lowest residual mass, which corroborates the porosity findings in Section 3.5.1. This demonstrates that the volumetric heating characteristics of microwaves and the selective heating of SiC construct a more uniform heat transfer field within the matrix, guiding the precise in situ growth of calcium carbonate crystals within the capillary pores. For the T90-t60 group, where the strength begins to decline, the weight loss peak remains sharp but narrows in width. This reflects that, although the crystallinity of the products is acceptable, the internal vapor pressure damage caused by excessive microwave exposure destroys the structural integrity of the matrix; thus, despite the abundance of products, they fail to provide effective structural support.

The results reveal that the reinforcement mechanism of the microwave synergistic system does not solely rely on increasing the total amount of carbonation products. The more critical pathway lies in optimizing the precipitation configuration and spatial distribution of products via the microwave thermal field. This mechanism achieves refined pore structure reconstruction without significantly altering the total product volume, thereby imparting superior mechanical performance to the steel slag matrix. Mechanical properties are governed not only by the degree of carbonation but also by factors such as the morphology, distribution characteristics, and interface bonding state of the carbonation products.

#### 3.5.3. Mineral Phase Analysis

**Figure 10:**
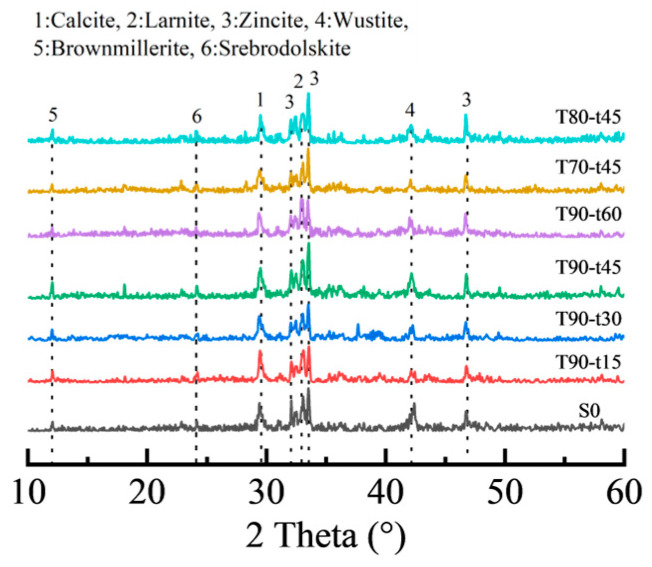
XRD patterns of carbonated steel slag under different treatment conditions.

Figure 10 illustrates the mineralogical phase variations of the samples under different processing conditions. The characteristic diffraction peak of calcite was observed at approximately 2θ = 29.4°. With increasing microwave heating temperature and duration, the calcite peak intensity generally increased, while the diffraction peaks associated with the original silicate phases (Larnite and β-C_2_S) exhibited a gradual decrease. These changes suggest that the microwave-assisted system may promote the dissolution of reactive calcium-bearing phases in steel slag and facilitate their subsequent carbonation reaction with CO_2_, leading to increased formation of carbonate products.

#### 3.5.4. FTIR Analysis

**Figure 11:**
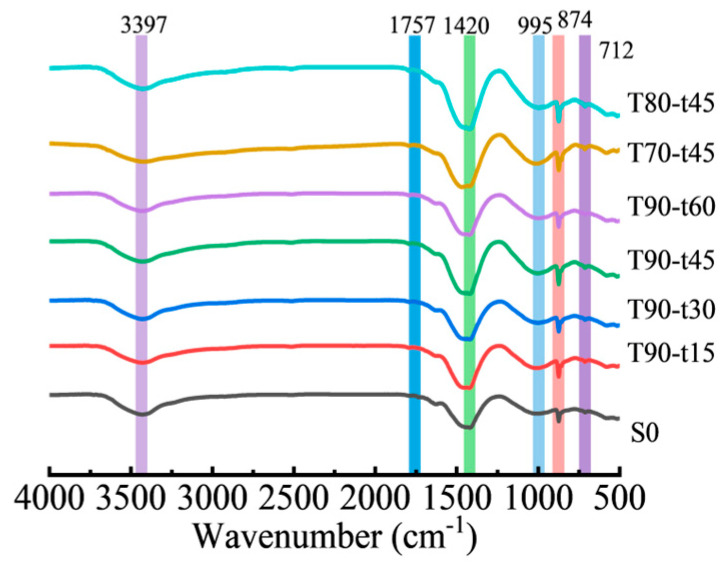
FTIR patterns of carbonated steel slag under different treatment conditions.

Figure 11 illustrates the positions of characteristic peaks in the FTIR spectrum: A broad, intense absorption peak at 3397 cm^−1^ corresponds to the stretching vibration of O-H bonds, originating from hydroxyl groups in physically adsorbed water, gel phases, Portlandite, and other hydrated oxides. A weaker absorption peak at 1757 cm^−1^ may be associated with C=O stretching vibrations.

The three most significant changes in the spectra occur in three key regions, corresponding to the primary carbonation reactions and silicate polymerization products. First, the sharp absorption peaks at 1420 cm^−1^ and 874 cm^−1^ correspond to the asymmetric stretching and out-of-plane bending vibrations of CO_3_^2−^, respectively, while the peak at 712 cm^−1^ corresponds to the in-plane bending vibration of CO_3_^2−^. These are all characteristic peaks of calcite-type calcium carbonate, the main carbonation product [39,48]. Second, the absorption band appearing near 995 cm^−1^ represents the asymmetric stretching vibration of Si-O-Si or Si-O bonds, which is a characteristic region for the silicate polymer network structure in gels.

For microwave heating, with the increase in temperature and time, the absorption peak features at 1420 cm^−1^ and 874 cm^−1^ show a trend of first becoming more pronounced and then weakening. Among all samples, the T90-t45 sample exhibits relatively more prominent characteristic absorptions at these two positions, which may suggest that under the selected processing parameters, the carbonation reaction proceeded more thoroughly, thereby forming more significant infrared characteristics of crystalline calcium carbonate. This result corresponds with the conclusions from Section 3.5.2 (maximum total weight loss in TG) and Section 3.5.3 (strongest calcite diffraction peak in XRD), jointly indicating from different analytical perspectives that this sample has a higher degree of carbonation.

Secondly, the microwave-assisted treated samples also display a clearer contour of the Si–O characteristic absorption peak at 995 cm^−1^, along with a shift toward a higher wavenumber, which is generally considered to be associated with an increased degree of polymerization of the silicon-oxygen framework. The above changes suggest that the introduction of potassium silicate, under microwave heating excitation, may have effectively participated in the reaction, facilitating the formation of silicate cementitious products such as C-S-H-like gels with a higher degree of polymerization. In the T90-t45 sample, both the peak at 995 cm^−1^ and the characteristic absorptions of calcium carbonate show relatively pronounced responses, which may reflect a trend of co-development and interweaving coexistence between the carbonation products and the silicate gel network. The construction of such a microstructure is expected to provide a favorable chemical bonding basis for the formation of a high-strength composite structure, potentially playing a positive microstructural supporting role in achieving superior compressive strength.

#### 3.5.5. Microstructural Analysis

**Figure 12:**
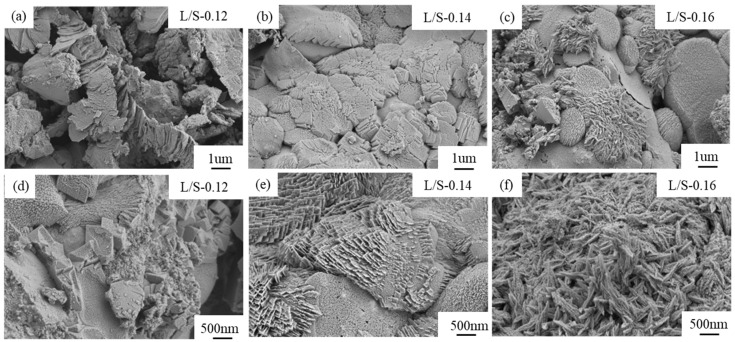
SEM images of carbonated steel slag specimens at different liquid-to-solid ratios, (**a**,**d**) L/S-0.12, (**b**,**e**) L/S-0.14, (**c**,**f**) L/S-0.16.

To reveal the influence mechanism of the microwave-induced synergistic system on the performance of carbonated steel slag blocks at the microscopic scale, scanning electron microscopy (SEM) and energy dispersive spectroscopy (EDS) were employed for morphological observation and micro-area elemental analysis. Figure 12 presents the SEM images of the specimens under different liquid-to-solid (L/S) ratios.

The L/S ratio determines the final morphology of the products by influencing the distribution of the reaction medium. At lower liquid content, the microstructure is characterized by relatively coarse and isolated crystalline phases, with incomplete pore filling. This indicates that nucleation density is limited and crystal growth dominates the reaction process. In contrast, higher liquid content results in refined crystal morphology, with the formation of densely packed granular and clustered structures. The increased nucleation density suppresses excessive crystal growth and leads to a more homogeneous distribution of carbonation products. Consequently, pore spaces are effectively filled, contributing to a denser microstructure. These observations demonstrate that liquid content regulates the balance between nucleation and crystal growth, thereby controlling the final microstructural configuration.

**Figure 13:**
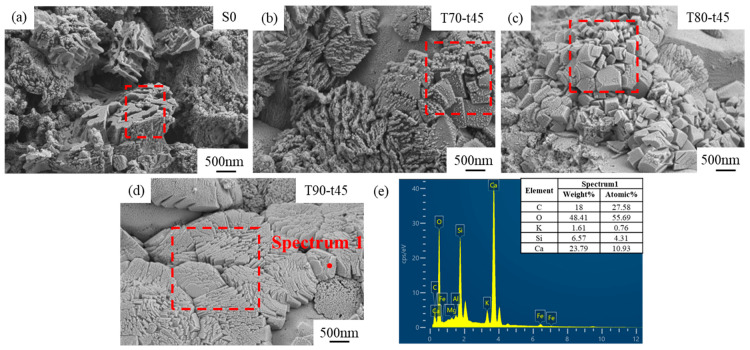
SEM and EDS images of carbonated steel slag specimens at different microwave heating temperatures, (**a**) S0, (**b**) T70-t45, (**c**) T80-t45, (**d**) T90-t45, (**e**) Spectrum 1.

**Figure 14:**
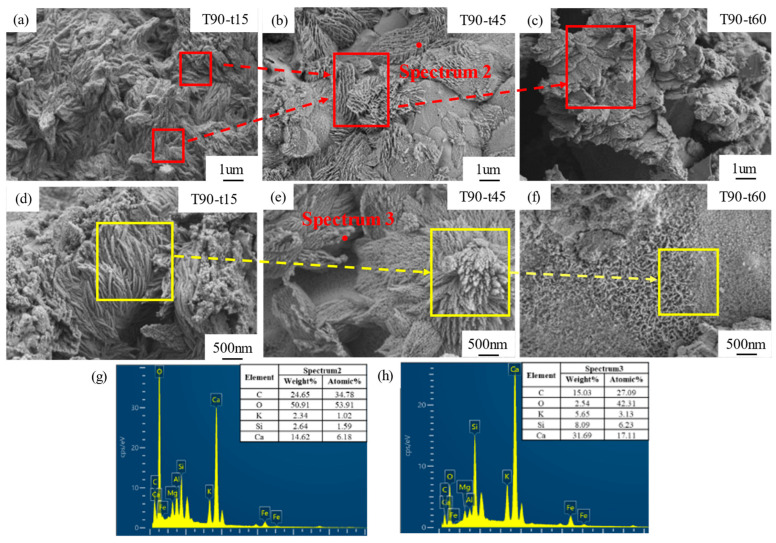
SEM and EDS images of carbonated steel slag specimens at different microwave heating times, (**a**,**d**) T90-t15, (**b**,**e**) T90-t45, (**c**,**f**) T90-t60, (**g**,**h**) Spectrum 2 and 3.

Figure 13 and Figure 14 present the SEM images of the specimens under different microwave treatment conditions.

The influence of microwave heating temperature on the evolution of the specimens’ micromorphology is illustrated in Figure 13a–d, with Figure 13e confirming that the cubic products are calcium carbonate. The S0 group, which lacks the microwave-SiC-potassium silicate synergistic system, exhibits a loose packing structure with distinct inter-particle voids. The carbonation products formed at the interface are sparse and fail to form an effective cementing network or a regular structure, which accounts for the lower macroscopic compressive strength of these samples.

As the microwave heating temperature increases from 70 °C to 90 °C, the structural densification of the matrix improves significantly. In the T70-t45 sample, particle surfaces begin to be covered by newly formed products, with flocculent gel phases filling the inter-particle spaces, leading to a decrease in porosity. When the temperature rises to 80 °C, distinct cubic crystals—typical of calcite—are observed nucleating and growing within the matrix. Simultaneously, flocculent gel products continue to form, interweaving with the calcite crystals. Upon reaching 90 °C, the microstructure exhibits a high degree of densification, characterized by a large volume of well-developed, interlaced calcium carbonate crystals. These crystals are regularly arranged, filling the original pores and interlocking with the gel products in a three-dimensional (3D) spatial configuration, achieving a homogenized gel-mineralized structure. This dense crystalline packing structure greatly enhances the bonding strength between aggregates, thereby increasing the overall strength of the sample. This indicates that the microwave-induced high-temperature field accelerates the nucleation kinetics of Ca^2+^ and CO_3_^2−^, promoting the transition of products toward the thermodynamically stable calcite phase and maximizing macroscopic strength.

The influence of microwave heating duration on the micromorphology evolution of the specimens is illustrated in Figure 14a–f. In the early heating stage, the specimens begin to form flocculent and flaky reaction products; however, their distribution is uneven, and a compact structure has not yet developed. As shown in Figure 14d, flocculent products can be clearly observed growing from the pores. As the heating duration increases to 45 min, the morphology evolves into unique “plate-like” layered stacks and “flower-like” clusters. This highly ordered arrangement significantly enhances the cohesive strength between crystals. EDS analysis suggests that the “plate-like” products are mainly calcium-carbonate-rich phases associated with minor silicate-rich gel-like products containing Ca, Si, and K elements, while the “flower-like” structures may correspond to K-rich silicate gel-like phases. However, it should be noted that EDS elemental analysis alone cannot conclusively identify specific amorphous gel structures such as C-S-H or K-S-H, and the above interpretations are therefore presented as inferred phase characteristics based on elemental composition and morphology.

When the duration is extended to 60 min, the originally dense crystal clusters disappear, replaced by an extensive network of porous structures. This loose network leads to a reduction in the effective load-bearing area and an increase in stress concentration points. Furthermore, micro-damage, such as the cracks and loose structural zones shown in Figure 14f, accounts for the observed decline in macroscopic compressive strength.

This phenomenon corroborates the MIP results discussed in Section 3.5.1, specifically the rebound in total porosity for the T90-t60 group. Excessive microwave heating generates intense internal vapor pressure and thermal stress mismatch, triggering a physical damage that compromises the previously formed dense structure.

**Figure 15:**
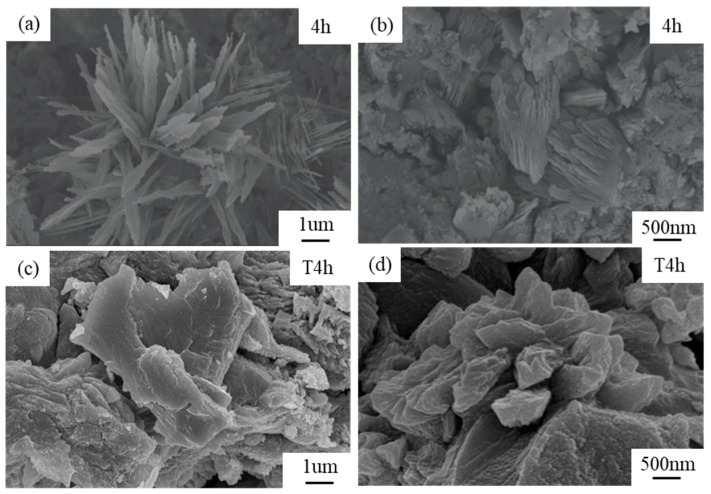
SEM images of carbonated steel slag specimens at different processing conditions, (**a**,**b**) 4h, (**c**,**d**) T4h.

To further investigate the microscopic driving effect of the microwave-assisted synergistic system on carbonation kinetics, this study compared the surface morphologies of specimens carbonated for 4 h under two conditions: the ambient pure water system (4h) and the microwave synergistic system (T4h). The corresponding findings are illustrated in Figure 15.

As shown in Figure 15, after 4 h of carbonation in the ambient pure water system, the surface of the steel slag particles exhibits distinct non-uniform growth characteristics. Loosely packed needle-like crystal structures are observed in certain areas, predominantly growing in radial and localized agglomerated forms with inconsistent orientations. These crystals are largely confined to localized active sites on the steel slag particles. Significant voids remain between the crystals, and a continuous, dense covering layer has failed to form. This indicates that, in the absence of external energy driving forces, the carbonation reaction is primarily limited by diffusion, resulting in a slow product-filling rate and a sparse structure.

Upon the introduction of the microwave-assisted synergistic system, the surface morphology of the 4 h carbonation samples underwent a significant qualitative transformation. The original loose needle-like crystal structures are markedly reduced, replaced by a large volume of flaky and layered products that are densely stacked and cover the particle surfaces. These flaky crystals exhibit clear boundaries and present a “shingle-like” overlapping stacking characteristic, which effectively achieves physical filling and structural bridging of the initial inter-particle pores. Compared to the rough and weakly bonded crystal interfaces in the pure water system, the products in the microwave-treated samples exhibit a more compact structural arrangement and a higher degree of densification.

This microscopic disparity elucidates the unique acceleration mechanism of the microwave-assisted system. By selectively heating SiC particles, microwave energy creates a localized high-temperature field at the matrix interface from the onset of the reaction, significantly enhancing the leaching concentration of Ca^2+^ and the dissolution rate of CO_2_. Combined with the activation effect of potassium silicate, this encourages the carbonation products to bypass the slow needle-like growth stage, evolving directly into the thermodynamically more stable and structurally denser layered calcite phase. This “early-stage morphological pre-optimization” establishes the critical material foundation for the subsequent surge in compressive strength during the carbonation process.

#### 3.5.6. Mechanism Analysis

**Figure 16:**
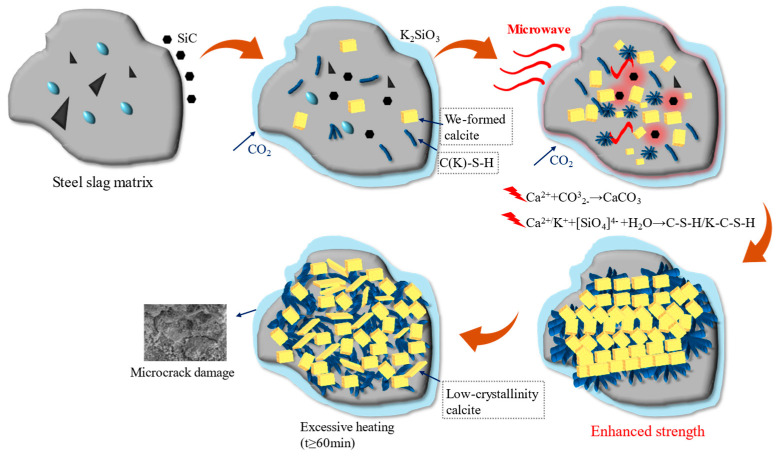
Schematic illustration of the microstructural evolution of steel slag during microwave-assisted carbonation.

The schematic diagram in Figure 16 elucidates the promotion mechanism of the synergistic effect between microwave heating and chemical additives on the carbonation of steel slag. Under microwave irradiation, SiC particles act as microwave-absorbing materials, creating a “hot zone” effect by converting electromagnetic energy into thermal energy. This generates localized high temperatures at the solid–liquid interface, achieving uniform volumetric heating of the steel slag mixture. This process significantly elevates the overall temperature of the reaction system and improves the uniformity of thermal distribution, overcoming the temperature gradient limitations of traditional heating methods that transfer heat from the surface inward. The uniformly enhanced temperature field significantly accelerates the leaching of active calcium ions from the steel slag, the dehydration and condensation of the potassium silicate gel, and the diffusion rate of CO_2_ gas within the pores.

Under optimized thermal-field and reaction-kinetic conditions, calcium-carbonate-rich products tend to nucleate more uniformly and gradually develop into relatively compact and regularly distributed crystalline structures. Meanwhile, silicate gel-like phases derived from potassium silicate may contribute to micropore filling and local structural densification. The combined effect of carbonate precipitation and gel-like product formation is suggested to promote pore refinement and matrix densification, thereby improving the macroscopic mechanical performance of the specimens.

However, when the microwave heating duration is further prolonged, the microstructure gradually becomes looser, accompanied by the appearance of irregular crystal morphology and localized defects. This structural deterioration may be associated with excessive thermal accumulation and internal vapor-pressure effects under prolonged microwave exposure, ultimately leading to the observed reduction in compressive strength.

## 4. Conclusions

This study systematically investigates the influence of a microwave-assisted system synergistic with SiC and potassium silicate on the carbonation behavior of steel slag, while providing an in-depth analysis of its microstructural evolution and reinforcement mechanisms.

1. The combined effects of enhanced thermal fields and alkali activation under microwave radiation conditions. Under the condition of microwave intervention at 90 °C for 45 min, the compressive strength of the specimens reached 48.82 MPa, representing a 44.7% increase compared to the ambient-temperature hydration-carbonation process. These findings indicate that the microwave-assisted synergistic system effectively overcomes the kinetic bottlenecks associated with slow traditional carbonation reactions.

2. Precision regulation of microstructure and porosity: The microwave-induced steel slag system achieves a structural transformation from “loose packing” to a “cross-linked dense” configuration. MIP analysis confirms that the average pore diameter was significantly refined from 97 nm in the conventional group to 43 nm. Microwave assistance facilitates the coupling of in situ growth and physical filling between the carbonation products (calcite) and alkali-activated products (C-S-H/K-S-H gels), constructing a more robust three-dimensional skeletal network.

3. Interface “hot zone” effect and morphological evolution mechanism: Mechanistic studies reveal that SiC, acting as a microwave absorber, generates a critical “hot zone” effect within the matrix. By accelerating Ca^2+^ leaching and CO_2_ diffusion, it alters the crystal growth trajectory. The carbonation products directly transform into a thermodynamically more stable and more compactly arranged layered calcite phase.

4. Energy input and damage threshold: The study identifies a distinct “time threshold” effect associated with microwave intervention. When the heating duration exceeds 45 min (e.g., 60 min), the sharp increase in internal steam pressure triggers physical damage. Coupled with uneven thermal stress distribution, this leads to the initiation of micro-cracks and a reduction in density, thereby causing a decline in mechanical performance.

In summary, the microwave-assisted synergistic carbonation strategy proposed in this study provides an efficient pathway for the high-value valorization of industrial steel slag. This method significantly shortens the curing duration and enables the effective mineral sequestration of CO_2_ while producing high-performance building materials, aligning with global “carbon peak and carbon neutrality” objectives for solid waste utilization. Future research should investigate the effects of varying microwave power and intervention timing on the carbonation of steel slag. This will facilitate a more comprehensive understanding of the mechanisms by which microwave treatment promotes carbonation, providing a solid theoretical framework to support its deployment at an industrial level.

## Figures and Tables

**Table 1:** Chemical composition of steel slag and SiC (wt%).

Composition	CaO	SiC	Fe_2_O_3_	SiO_2_	MgO	Al_2_O_3_	MnO	P_2_O_5_	TiO_2_	Others
Steel slag	41.66	/	30.80	11.70	4.21	3.46	3.31	2.26	0.97	1.21
SiC	/	96.28	0.78	/	/	/	/	/	/	2.94

**Table 2:** Sample name treated under the microwave-SiC-K_2_SiO_3_ composite system.

Temperature/°C	Time/mins			
	15	30	45	60
70	T70-t15	T70-t30	T70-t45	T70-t60
80	T80-t15	T80-t30	T80-t45	T80-t60
90	T90-t15	T90-t30	T90-t45	T90-t60

**Table 3:** Pore structure data of carbonated steel slag under different treatment conditions.

Sample Name	SS	S0	T90-t45	T90-t60
Porosity (%)	27.97	24.99	23.37	25.1
Bulk Density (g/mL)	2.22	2.16	2.20	2.21
Apparent Density (g/mL)	3.07	2.88	2.87	2.96
Average pore diameter (nm)	770	97	43	48

## Data Availability

The original contributions presented in this study are included in the article. Further inquiries can be directed to the corresponding author.
